# New Promoters for Metabolic Engineering of *Ashbya gossypii*

**DOI:** 10.3390/jof7110906

**Published:** 2021-10-26

**Authors:** Gloria Muñoz-Fernández, Javier-Fernando Montero-Bullón, José Luis Revuelta, Alberto Jiménez

**Affiliations:** Metabolic Engineering Group, Department of Microbiology and Genetics, University of Salamanca, 37007 Salamanca, Spain; gmf@usal.es (G.M.-F.); jfmonbul@usal.es (J.-F.M.-B.); revuelta@usal.es (J.L.R.)

**Keywords:** *Ashbya gossypii*, gene expression, promoter, luciferase, metabolic engineering

## Abstract

*Ashbya gossypii* is a filamentous fungus that is currently exploited for the industrial production of riboflavin. In addition, metabolically engineered strains of *A. gossypii* have also been described as valuable biocatalysts for the production of different metabolites such as folic acid, nucleosides, and biolipids. Hence, bioproduction in *A. gossypii* relies on the availability of well-performing gene expression systems both for endogenous and heterologous genes. In this regard, the identification of novel promoters, which are critical elements for gene expression, decisively helps to expand the *A. gossypii* molecular toolbox. In this work, we present an adaptation of the Dual Luciferase Reporter (DLR) Assay for promoter analysis in *A. gossypii* using integrative cassettes. We demonstrate the efficiency of the analysis through the identification of 10 new promoters with different features, including carbon source-regulatable abilities, that will highly improve the gene expression platforms used in *A. gossypii*. Three novel strong promoters (*P_CCW12_*, *P_SED1_*, and *P_TSA1_*) and seven medium/weak promoters (*P_HSP26_*, *P_AGL366C_*, *P_TMA10_*, *P_CWP1_*, *P_AFR038W_*, *P_PFS1_*, and *P_CDA2_*) are presented. The functionality of the promoters was further evaluated both for the overexpression and for the underexpression of the *A. gossypii* *MSN2* gene, which induced significant changes in the sporulation ability of the mutant strains.

## 1. Introduction

*Ashbya gossypii* is a filamentous hemiascomycete of the Saccharomycetaceae family with industrial relevance since it is a natural overproducer of riboflavin (vitamin B2). Indeed, engineered strains of *A. gossypii* are currently exploited as microbial factories for the industrial production of riboflavin [1,2]. Besides the utilization of *A. gossypii* for riboflavin production, during the last decade, this fungus has emerged as a potential microbial factory for the production of other purine-related metabolites such as nucleosides and folic acid [3,4], and other bioproducts with industrial interest such as proteins, biolipids, and gamma-lactones [5,6,7]. In addition, industrial residues and by-products such as lignocellulosic hydrolysates, molasses, and crude glycerol have been documented as efficient carbon sources for *A. gossypii* [8,9].

The importance of *A. gossypii* in industry owes itself to certain beneficial aspects regarding bioprocessing and metabolic engineering. First, the bioprocessing is cost-effective since *A. gossypii* has the ability to grow using low-cost substrates and the downstream processing is inexpensive [10]. Second, there is a large and efficient molecular toolbox for the genomic manipulation of the fungus, including gene-targeting methods, heterologous expression platforms, or CRISPR/Cas9/Cas12 adapted systems [6,11,12,13].

Systems metabolic engineering applied to produce complex, high added-value compounds requires well-performing gene expression tools. In *S. cerevisiae*, the production of the antimalarial artemisinic acid required seven engineering steps to deregulate the mevalonate pathway and redirect metabolic flux towards artemisinic acid [14]. In *Yarrowia lipolytica*, 30 copies of nine different genes were used to produce high levels of the omega-3 fatty acid eicosapentaenoic acid [15]. More recently, the yeast *Pichia pastoris* has been engineered to grow on CO_2_ using up to eight heterologous genes [16]. All those successful approaches are based on the availability of a large collection of constitutive and inducible promoters that enable the use of several different expression platforms.

The manipulation of *A. gossypii* is restricted to genomic integrations since no stable plasmids exist for this fungus. Hence, gene deletions, as well as gene underexpression and overexpression, are carried out using integrative cassettes [17,18]. Likewise, heterologous gene expression is accomplished by using single/multiple overexpression platforms with a single integrative cassette [13]. The utilization of those molecular tools in *A. gossypii* enabled the simultaneous modification/engineering of up to eight genes for the production of polyunsaturated fatty acids from glucose [13] and microbial oils from xylose-based residues [8]. However, the recurrent utilization of the same strong promoter for gene overexpression can affect the fitness of the engineered strains, probably due to the presence of several identical sequences, and, thereby, limit the number of manipulations to be introduced in the genome. In this regard, to develop optimized expression platforms in *A. gossypii*, the identification of native promoters covering different expression patterns will highly improve the molecular toolbox for this fungus.

While several strong native constitutive promoters are used in this fungus such as *P_GPD1_*, *P_TEF_*, *P_PGK1_*, and *P_ADH1_* [6,13], only one weak native promoter from *AgRIB7* has been reported for gene underexpression [17]. Additionally, a few regulatable promoters have been described in *A. gossypii*, such as *P_MET3_* [19], for methionine-dependent downregulation, or the thiamine-repressible *S. cerevisiae THI13* promoter [20].

In this context, plasmid-based GFP- and lacZ-reporter assays were previously described for promoter analysis in *A. gossypii* [19]. However, as mentioned above, plasmids are not fully stable in the multinucleated syncytium of *A. gossypii*, and, moreover, the variability in the plasmid copy number can add experimental inaccuracy. Hence, the development of new methods for promoter analysis in *A. gossypii* using integrative cassettes is desirable.

In this work we present a method for promoter analysis in *A. gossypii* using integrative cassettes based on the mammalian Dual Luciferase Reporter (DLR) Assay, which allows the sequential quantitative measurement of two luciferase activities (*Renilla* and firefly luciferases) in a single protein extract, thus conferring accuracy and reproducibility to the system. We report 10 new promoters covering a wide range of promoter activity with important potential applications for metabolic engineering of *A. gossypii*.

## 2. Materials and Methods

### 2.1. Ashbya gossypii Strains and Growth Conditions

The *A. gossypii ATCC 10,895* strain was used as the wild-type strain. The *A. gossypii* strains generated in this study are listed in Table S1. *A. gossypii* liquid cultures were initiated with spores (10^6^ spores/L) and carried out at 28 °C and 200 rpm using MA2-rich medium (2% bactopeptone, 0.2% yeast extract, 0.06% myo-inositol, and pH 6.8) with the indicated carbon source, either 2% glucose or 2% oleic acid (OA) plus 0.5% glucose. *A. gossypii* transformation, spore isolation, and sporulation conditions were as described in [21]. A concentration of 250 mg/L for geneticin (G418) (Gibco-BRL, Waltham, MA, USA) was used where indicated.

### 2.2. Assembly and Genomic Integration of the Cassettes for Renilla and Firefly Luciferase Expression

The integrative cassettes used in this work were assembled using a Golden Gate method, as described in [13]. The integrative cassettes comprised recombinogenic flanks targeting either the *ADR304W* or *AGL034C* loci, a *loxP-KanMX-loxP* (G418^R^) marker, and the transcriptional units for either *Renilla* or firefly luciferase expression (Table S2—NCBI accession numbers: *Renilla* luciferase, AAB82577.1; firefly luciferase, AAA29795.1). For the expression of the *Renilla* luciferase, the strong constitutive promoter *P_GDP1_* was used; for the expression of the firefly luciferase, different promoter sequences were used. The CDS of *Renilla* and firefly luciferases were PCR-amplified from the pRL-SV40 (Promega; NCBI accession number: AF025845.2) and pAP1-luc (Stratagene; NCBI accession number: AF053698.1), respectively. In addition, the intergenic promoter sequences were PCR-amplified from *A. gossypii* genomic DNA. Those PCR primers contained a *Bsa*I recognition site and 4-nucleotide overhangs for the Golden Gate assembly (see Table S3 for primer sequences). All the modules were assembled into a destination vector that contains a spectinomycin resistance marker (Figure S1). The assembled plasmids were selected in spectinomycin/kanamycin-containing LB plates and were confirmed by restriction analysis and DNA sequencing. The final integrative cassettes were isolated by *Sap*I digestion and used for *A. gossypii* transformation.

Spores of *A. gossypii* were transformed with the integrative cassettes, and positive primary heterokaryotic clones were selected in G418-containing medium. Homokaryotic clones were obtained by sporulation of the primary transformants. The correct genomic integration of each integrative cassette was confirmed by analytical PCR followed by DNA sequencing. The transient expression of a Cre recombinase enabled the *loxP-kanMX-loxP* marker to be eliminated and reused, as described elsewhere [22].

### 2.3. Dual Luciferase Reporter (DLR) Assay for Promoter Analysis

A DLR assay system (Promega, Madison, WI, USA) was used for promoter analysis. Flask cultures for luciferase assays were initiated with 10^5^ spores and carried out in 30 mL of MA2 rich medium at 28 °C for 40 h. A mycelial biomass of 100 µL was harvested, washed twice with 200 µL of PBS and resuspended in 100 µL of Passive lysis Buffer from the DLR assay kit (Promega). Cell disruption was carried out with glass beads (0.5 mm) by vortexing 4 times for 15 s, each time keeping the cells on ice for 1 min between vortexing. The luciferase assay was performed following the manufacturer’s instructions of the DLR assay kit (Promega). Luminescence of both *Renilla* and firefly luciferases were measured sequentially using a Varioskan microtiter plate reader (Thermo Scientific, Waltham, MA, USA). The results were represented as a ratio of the luciferase activity of firefly and *Renilla* (ratio Fluc/Rluc), which allows for a normalization of the results with an internal control.

### 2.4. Construction and Integration of MSN2 Expression Cassettes

Different integrative cassettes targeting the *AgMSN2* (*ABR089C*) gene were PCR-amplified from the firefly expression cassettes described above using the primers listed in Table S3. Each cassette was integrated upstream of the ATG initiator codon of *AgMSN2* by homologous recombination using recombinogenic flanks that were included in the primer sequences. The expression cassettes comprised a different promoter sequence and the *loxP-KanMX-loxP* (G418^R^) selectable marker. Genomic integration of the expression cassettes was confirmed by analytical PCR.

### 2.5. Quantitative Real-Time PCR

Quantitative real-time PCR (qRT-PCR) was performed with a LightCycler 480 real-time PCR instrument (Roche, Basel, Switzerland), using SYBR Green I master mix (Roche) following the manufacturer’s instructions. Total RNA samples were prepared as described in [23]. cDNA was synthesized using the Transcriptor First Strand cDNA Synthesis Kit (Roche). Primer sequences are listed in Table S3. qRT-PCR reactions were performed in duplicate and in at least two independent experiments. Quantitative analyses were carried out using the LightCycler 480 software. The mRNA level of the target genes was normalized to that of *AgUBC6* and was calculated using the 2^−∆∆Ct^ method [24].

### 2.6. RNAseq

The *A. gossypii* WT strain was grown in 50 mL of MA2 flask cultures for 72 h. Mycelia were harvested, and total RNA samples were prepared as described in [23]. Total RNA was used for Illumina Hiseq 2000 sequencing. RNAseq was performed by Macrogen (Seoul, South Korea). Raw data were processed, and reads were aligned to the *A. gossypii* reference genome using Geneious R11 software 10.0.5. The expression level for each coding sequence was calculated based on the normalized FPKM (fragments per kb/million mapped reads).

### 2.7. Sporulation Analysis

Mycelia of selected *A. gossypii* strains were cultured onto sporulation (SPA, sporulation of Ashbya) media plates for 4 days at 28 °C and spores were isolated from 100 mg of mycelia that were scraped out from each plate. Serial dilutions of the spore preparations were performed in 0.01% Triton X-100, plated on MA2 medium, and incubated at 28 °C until colonies appeared. Additionally, the number of spores from 10^−2^ dilutions were counted using a Neubauer cell chamber. Data are expressed as the number of spores/g of mycelium of each strain.

## 3. Results and Discussion

### 3.1. Adaptation of a Luciferase Reporter Assay for Promoter Analysis in A. gossypii

**Figure 1:**
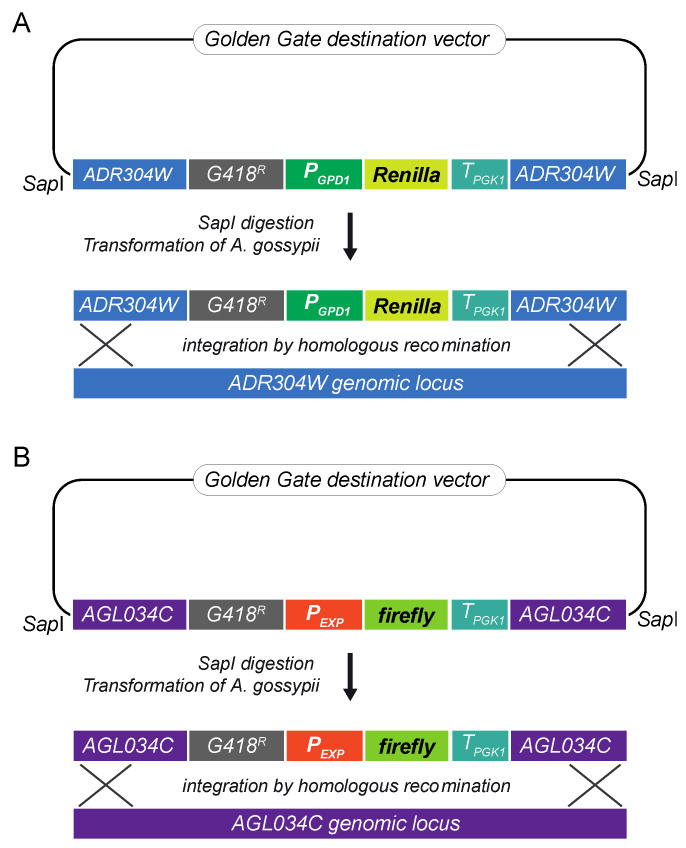
Assembly of the DLR (Dual Luciferase Reporter) system adapted for *A. gossypii*: (**A**) Integrative cassette for the expression of the *Renilla* luciferase under the control of the *P_GPD1_* promoter; (**B**) Integrative cassette for the expression of the firefly luciferase under the control of the different experimental promoters. All the modules were assembled into a destination vector following a Golden Gate method. The integrative cassettes were obtained by *Sap*I digestion and used for *A. gossypii* transformation. Genomic integration occurred by homologous recombination.

A luciferase reporter assay was designed to evaluate the ability of different promoter sequences to drive gene expression in *A. gossypii*. Since episomic vectors are not fully stable in *A. gossypii*, the promoter activities were assessed using genomic integrative cassettes. The coding sequences of the *Renilla* (*Renilla reniformis*) and firefly (*Photinus pyralis*) luciferases were used in a dual luciferase reporter assay to improve experimental accuracy. Hence, two integrative cassettes were assembled: an integrative module for the expression of the *Renilla* luciferase was used as the internal control, and an integrative cassette for the firefly luciferase was used as the experimental reporter. Each genomic integrative cassette comprised recombinogenic flanks, a *loxP-KanMX-loxP* (G418^R^) marker, the promoter sequence, the reporter luciferase CDS, and the terminator sequence of *PGK1* (Figure 1). The recombinogenic flanks target the *ADR304W* and *AGL034C* loci in the integrative cassette for *Renilla* and firefly, respectively. The disruption of either *ADR304W* or *AGL034C* loci does not affect growth in *A. gossypii*, as previously described in [25]. In this work, the strong promoter *P_GPD1_* was chosen as the internal control. The integrative cassettes were assembled following a Golden Gate modular cloning system adapted for *A. gossypii*, (Figure 1) [13].

**Figure 2:**
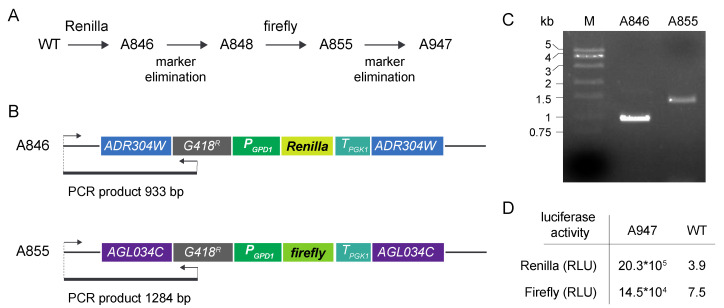
Construction of the DLR control strains: (**A**) Pipeline of the construction of the A947 control strain harboring both *Renilla* and firefly luciferases under the control of *P_GPD1_*; (**B**) Scheme for the ADR304W and AGL034C loci after genomic integration of the *Renilla* and firefly integrative cassettes; the strategy for the analytical PCRs is depicted; (**C**) Analytical PCR of the *A. gossypii* strains containing the *Renilla* (A846) and the firefly (A855) expression cassettes; (**D**) *Renilla* and firefly luciferase activities of the final A947 control strain and the WT expressed as Relative Light Units (RLU).

For the construction of the internal control strain, a wild-type strain of *A. gossypii* was first transformed with the integrative module for *Renilla* luciferase expression to generate strain A846 (Figure 2A). The G418^R^ marker was eliminated in the A846 strain by Cre recombinase expression to generate control strain A848. This strain was transformed with the corresponding cassette for firefly expression to obtain strain A855. The integration of the expression cassettes in the target loci was verified by analytical PCR (Figure 2B,C). After marker elimination, an initial strain, named A947, was obtained (Figure 2A), which was equipped with both the *Renilla* and firefly expression cassettes containing the strong promoter *P_GPD1_*. The expression of both *Renilla* and firefly luciferases from *P_GPD1_* in strain A947 was analyzed using the dual luciferase assay (Figure 2D), thus confirming the functionality of the system. The luciferase assay was carried out using total protein extracts from the A947 strain grown in MA2 rich medium with 2% glucose as the carbon source for 40 h. The activities of *Renilla* and firefly luciferases were measured as described in the Materials and Methods section.

### 3.2. Dual luciferase Assay of Selected Promoter Sequences

To validate the system for promoter analysis in *A. gossypii*, the intergenic sequences upstream the ATG codon from different genes of *A. gossypii* were challenged for their ability to drive the expression of the firefly luciferase. The promoter sequences were chosen according to our preliminary RNAseq data (unpublished results) and included a total of 10 genes that showed either higher, similar, or lower expression levels than the *GPD1* (*TDH3*) gene (Table 1). The length of the selected promoter sequences extended from 176 bp to 1000 bp, depending on each intergenic sequence (Table S4).

The promoter sequences were PCR amplified using specific primers (Table S3) that contained a *Bsa*I recognition site and 4-nucleotide overhangs for the assembly of the integrative cassettes using a Golden Gate method. The control strain A848, containing the *Renilla* expression cassette (Figure 1A), was then transformed with the integrative cassettes containing the experimental reporter firefly luciferase under the control of different promoter sequences (Figure 1B). The integration of each expression cassette in the *ADR304W* locus was confirmed by analytical PCR (not shown). Prior to the luciferase assay, the G418^R^ marker was eliminated in all the engineered strains (Table S1).

**Figure 3:**
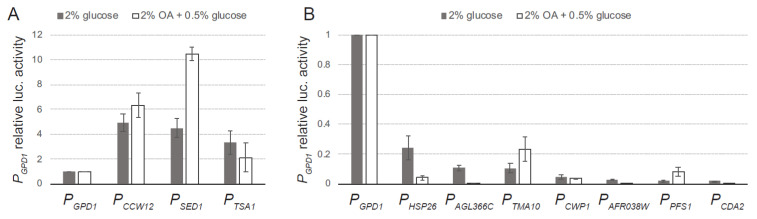
Luciferase assay of selected promoters: (**A**) DLR assay of strong promoters; (**B**) DLR assay of medium/weak promoters. The luciferase assays were carried out from cultures containing either 2% glucose (grey bars) or 2% oleic acid (OA) + 0.5% glucose (white bars). Data are the average of three independent experiments, performed in duplicate, and are expressed as relative luciferase activities with respect to *P_GPD1_* activity.

The activities of *Renilla* and firefly luciferases were measured in all the generated strains grown in MA2 rich medium with 2% glucose as the carbon source for 40 h. The promoter activities of the analyzed sequences were compared with the activity of the strong promoter *P_GPD1_*. Our data showed that three sequences (*P_SED1_*, *P_CCW12_*, and *P_TSA1_*) provided a strong promoter activity, between 3 to 5 times higher than that of the *P_GPD1_* (Figure 3A). In addition, the intergenic sequences corresponding to the genes *HSP26*, *AGL366C*, *TMA10*, *CWP1*, *AFR038W*, *CDA2*, and *PFS1* were also able to drive the expression of the firefly luciferase, thereby confirming their promoter activity. These promoters showed a wide range of transcriptional activity from about 4 to 55 times lower than that of the *P_GPD1_* (Figure 3B). Hence, the promoter sequences analyzed can be categorized into three classes according to their promoter activity: strong, medium, and weak promoters (Figure S2), offering a dynamic range of promoter activity. Remarkably, while the genes *TMA10* and *CWP1* exhibited higher transcription level than *GPD1* (Table 1), their corresponding intergenic sequences showed very low promoter activity. Indeed, according to our results, the promoter activity of the intergenic sequences used in the analysis does not correlate directly with the transcription level of their corresponding genes (Figure S2), indicating that additional *CIS*-acting elements, either positive or negative, must be involved in the transcriptional regulation of the chosen genes. Consequently, the identification of new promoters for metabolic engineering cannot solely rely on gene expression analyses, but must be addressed by using efficient tools for promoter analysis, which can also be applied to uncover *CIS*-acting elements within a promoter sequence.

The identification of inducible promoters with carbon source responsiveness also constitutes an important issue for metabolic engineering approaches. For example, the bioproduction of riboflavin using *A. gossypii* industrial strains employs low-cost oil as a carbon source [26], and, therefore, it is of interest to find regulatable promoters that can be either induced or repressed in oil-containing media. In this regard, we decided to evaluate the promoter activity of the selected sequences in cultures containing 2% oleic acid (OA) plus 0.5% glucose. The addition of glucose promotes the rapid germination of the spores in the early stages of cultures, as described in [27]. Hence, the luciferase assay was performed from cultures grown for 40 h, when the OA is essentially the only carbon source. Our data showed that several promoters exhibited substantial differences in their relative luciferase activity depending on the carbon source. While the promoter activity of *P_SED1_*, *P_TMA10_*, and *P_PFS1_* was significantly induced in OA-containing media, the activity of *P_HSP26_* and *P_AGL366C_* was repressed compared with the cultures using glucose as the only carbon source (Figure 3). Thus, the promoter analysis used in this work represents an efficient tool for the identification of promoter sequences with different features, including regulatable abilities. Moreover, it is also possible to further characterize the regulatory properties of each promoter by a genetic dissection of the promoter *CIS*-acting elements. *A. gossypii*, which is mainly considered a microbial factory, also represents an adequate model organism in cell biology studies regarding polarized growth and ploidy [28,29]; therefore, the availability of new molecular tools will strongly benefit *A. gossypii* research.

### 3.3. In Vivo Analysis of New Promoter Sequences

**Figure 4:**
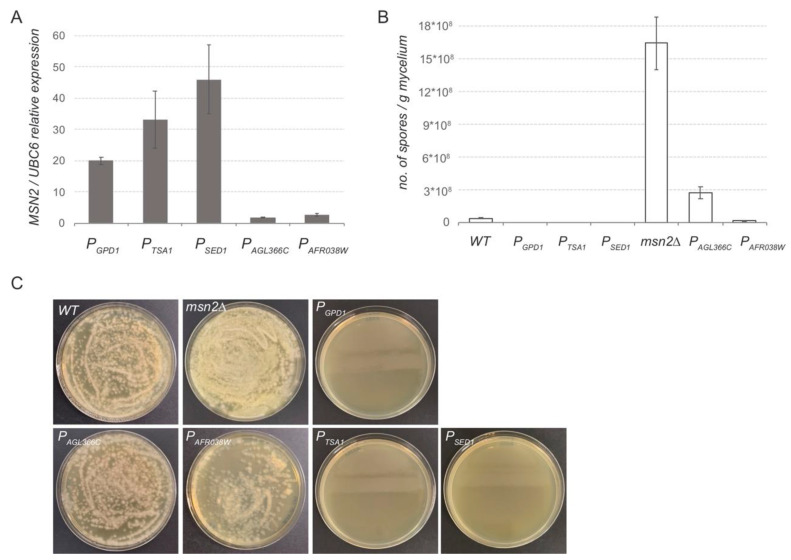
In vivo analysis of promoter sequences: (**A**) qPCR gene expression analysis of *MSN2* controlled by different promoter sequences. Transcription levels of *MSN2* were normalized using the *A. gossypii UBC6* gene as a reference. The results are the means of two independent experiments performed in duplicate. (**B**) Sporulation analysis of *A. gossypii* strains where *MSN2* is controlled by different promoter sequences. The wild-type and *msn2∆* strains are included as references. Spores were obtained from 100 mg of mycelia from the different strains grown in SPA media. Data were obtained from three independent experiments. (**C**) Dilutions of the spore preparations were plated onto MA2 media for visualization.

Next, we wanted to confirm the results obtained from the luciferase assay in modifying the expression of an *A. gossypii* endogenous gene. The *MSN2* gene was selected because the alteration on its expression level triggers major changes in the sporulation ability of *A. gossypii* [28]. We previously observed that the overexpression of *MSN2* nearly abolished the sporulation ability of *A. gossypii*, whereas the *msn2∆* strain showed a significant increase in the sporulation capacity with respect to the wild-type strain (unpublished results). Hence, integrative expression cassettes including the promoter sequences of *P_SED1_*, *P_TSA1_*, *P_AGL366C_*, and *P_AFR038W_* were PCR-amplified using specific primers that provided recombinogenic flanks targeting the *MSN2* gene of *A. gossypii*. Homokaryotic strains were obtained and the *loxP-KanMX-loxP* marker was eliminated in all the engineered strains. The expression level of the *MSN2* gene was measured by qPCR in all the strains generated (Figure 4A). Our data showed that both *P_SED1_* and *P_TSA1_* can be considered bona fide strong promoters that were able to provide a significantly higher expression level than the well-known *P_GPD1_*, which is generally used for gene overexpression. In addition, both *P_AGL366C_* and *P_AFR038W_* exhibited about 10 times less promoter strength than that of *P_GPD1_*, again confirming our previous results with the luciferase assay.

As mentioned above, the expression level of *MSN2* highly determines the sporulation ability of *A. gossypii*. Consequently, the sporulation rate was analyzed in the aforementioned strains, where the transcription of *MSN2* is driven by different promoter sequences. Both the *MSN2*-overexpressing strain (*P_GPD1_*) and the *msn2∆* strain were used as control strains. Our data showed that the utilization of strong promoters such as *P_GPD1_*, *P_SED1_*, and *P_TSA1_*, which provided high levels of *MSN2* expression, completely abolished the sporulation capacity of *A. gossypii* (Figure 4B,C). In contrast, the expression of *MSN2* under the control of the weak promoters *P_AGL366C_* and *P_AFR038W_* maintained the sporulation ability within the range of the WT strain, but far below the sporulation rate of the *msn2∆* strain (Figure 4B,C).

Taken together, our results demonstrate a good correlation between the data obtained with the luciferase assays and the in vivo analysis. Hence, the promoters presented in this work will assist the implementation of multiple expression platforms for metabolic engineering in *A. gossypii*. Three new strong promoters (*P_CCW12_*, *P_SED1_*, and *P_TSA1_*) can be applied to gene overexpression platforms. Additionally, *P_SED1_* can be considered an effective option for gene overexpression in lipid-based culture media. In contrast, seven novel promoters (*P_HSP26_*, *P_AGL366C_*, *P_TMA10_, P_CWP1_*, *P_AFR038W_*, *P_PFS1_*_,_ and *P_CDA2_*) are available for gene underexpression, including lipid-dependent regulatory properties for *P_HSP26_*, *P_AGL366C_*, *P_TMA10_*, and *P_PFS1_*. The adapted DLR system represents an efficient molecular tool for promoter analysis in *A. gossypii* beyond its biotechnological applications. For example, the OA-regulatable promoters can be used for the identification of oleate response elements (ORE), which participate in oleate induction [30]. In this regard, promoter tools have been described in other filamentous fungi such as *Trichoderma reesei*, *Aspergillus* spp., and *Penicillium chrysogenum*, which are also used for the production of industrially relevant metabolites [31,32,33,34], highlighting the importance of the identification of native promoters that can be used for the development of efficient expression platforms.

## Figures and Tables

**Table 1:** Selected promoter sequences used in the present study.

Gene	Standard Name	*S. cerevisiae* Homolog	RNAseq FKPM *	GO Description	Promoter Length (bp)
*AGR049W*	*CCW12*	*YLR110C*	102847.2	Cell wall mannoprotein	432
*AFR505C*	*TMA10*	*YLR327C*	71752.9	Protein of unknown function that associates with ribosomes	258
*ACR272C*	*CWP1*	*YKL096W*	28833.9	Cell wall mannoprotein that localizes to birth scars of daughter cells	950
*AER312W*	*TSA1*	*YML028W*	25764.9	Thioredoxin peroxidase. Panther family PTHR10681	255
*AER031C*	*GDP (TDH3)*	*YGR192C*	15480.9	Glyceraldehyde-3-phosphate dehydrogenase. Panther family PTHR10836	373
*AGL366C*		No homolog	14317.3		672
*AGR138W*	*SED1*	*YDR077W*	13184.5	Major stress-induced structural GPI-cell wall glycoprotein. Panther family PTHR35523	711
*ADL036C*	*CDA2*	*YLR308W*	12871.6	Chitin deacetylase. Panther family PTHR10587	325
*AGR408W*	*HSP26*	*YBR072W*	5158.0	Small heat shock protein (sHSP) with chaperone activity	1000
*AFR038W*		*YHR138C*	2681.4	Protein of unknown function	202
*AFR132C*	*PFS1*	*YHR185C*	72.8	Sporulation protein required for prospore membrane formation	176

* FPKM, Fragments per kilobase per million mapped reads.

## Data Availability

Data is contained within the article or supplementary material.

## Supplementary Materials

The following are available online at https://www.mdpi.com/article/10.3390/jof7110906/s1: Figure S1: Map of the Golden Gate destination vector; Figure S2: Comparison between the gene expression level and the promoter activity of the selected sequences; Table S1: *A. gossypii* strains used in this study; Table S2: Relevant sequences of the integrative cassettes; Table S3: List of primers used in this study; Table S4: Intergenic sequences used for the promoter analyses.
