# Supplementary material for: New Promoters for Metabolic Engineering of Ashbya gossypii
Source: J Fungi (Basel). 2021 Oct 26;7(11):906. doi: 10.3390/jof7110906 (PMC8618306; doi:10.3390/jof7110906)
Supplement: Supplementary file 1 [file jof-07-00906-s001.zip › Figure S2.pdf]

Muñoz-Fernández et al, 2021. Figure S2

Promoter	P_{GPD} relative promoter activity	
P_{CCW12}	4.96	
P_{SED1}	4.50	Strong promoters
P_{TSA1}	3.33	
P_{HSP26}	0.24	
$P_{AGL366C}$	0.11	Medium promoters
P_{TMA10}	0.11	
P_{CWP1}	0.05	
$P_{AFR038W}$	0.03	
P_{PFS1}	0.02	Weak promoters
P_{CDA2}	0.02	

Gene	*GPD* relative gene expresion	P_{GPD} relative promoter activity
CCW12	6.64	4.96
TMA10	4.63	0.11
CWP1	1.86	0.05
TSA1	1.66	3.33
AGL366C	0.92	0.11
SED1	0.85	4.50
CDA2	0.83	0.02
HSP26	0.33	0.24
AFR038W	0.17	0.03
PFS1	0.005	0.02
