# Supplementary material for: New Promoters for Metabolic Engineering of Ashbya gossypii
Source: J Fungi (Basel). 2021 Oct 26;7(11):906. doi: 10.3390/jof7110906 (PMC8618306; doi:10.3390/jof7110906)
Supplement: Supplementary file 1 [file jof-07-00906-s001.zip › Table S1.pdf]

Table S1. *A. gossypii* strains used in this study.

**Strain**	**Genotype**	**Source**
WT	Wild type (ATCC 10895)	our lab stock
A751	*MSN2*Δ	our lab stock
A783	P_{GPD1}-*MSN2*	our lab stock
A846	*adr304w::loxP-kanMX-loxP-*P_{GPD1}*-Renilla-*T_{PGK1}; Gt^R	this work
A848	*adr304w::*P_{GPD1}*-Renilla-*T_{PGK1}	"
A855	*adr304w::*P_{GPD1}*-Renilla-*T_{PGK1}*; agl034c::loxP-kanMX-loxP-*P_{GPD1}*-Firefly-*T_{PGK1}; Gt^R	"
A947	*adr304w::*P_{GPD1}*-Renilla-*T_{PGK1}*; agl034c::*P_{GPD1}*-Firefly-*T_{PGK1}	"
A986	*adr304w::*P_{GPD1}*-Renilla-*T_{PGK1}*; agl034c::*P_{SED1}*-Firefly-*T_{PGK1}	"
A987	*adr304w::*P_{GPD1}*-Renilla-*T_{PGK1}*; agl034c::*$P_{AGL366C}$*-Firefly-*T_{PGK1}	"
A988	*adr304w::*P_{GPD1}*-Renilla-*T_{PGK1}*; agl034c::*P_{TMA10}*-Firefly-*T_{PGK1}	"
A989	*adr304w::*P_{GPD1}*-Renilla-*T_{PGK1}*; agl034c::*P_{PFS1}*-Firefly-*T_{PGK1}	"
A990	*adr304w::*P_{GPD1}*-Renilla-*T_{PGK1}*; agl034c::*P_{HSP26}*-Firefly-*T_{PGK1}	"
A991	*adr304w::*P_{GPD1}*-Renilla-*T_{PGK1}*; agl034c::*P_{TSA1}*-Firefly-*T_{PGK1}	"
A992	*adr304w::*P_{GPD1}*-Renilla-*T_{PGK1}*; agl034c::*P_{CWP1}*-Firefly-*T_{PGK1}	"
A993	*adr304w::*P_{GPD1}*-Renilla-*T_{PGK1}*; agl034c::*P_{CCW121}*-Firefly-*T_{PGK1}	"
A997	*adr304w::*P_{GPD1}*-Renilla-*T_{PGK1}*; agl034c::*P_{AFR038}*-Firefly-*T_{PGK1}	"
A998	*adr304w::*P_{GPD1}*-Renilla-*T_{PGK1}*; agl034c::*P_{CDA2}*-Firefly-*T_{PGK1}	"
A1121	P_{TSA1}*-MSN2*	"
A1127	P_{SED1}*-MSN2*	"
A1128	$P_{AGL366C}$*-MSN2*	"
A1129	$P_{AFR038W}$*-MSN2*	"
