# Supplementary material for: New Promoters for Metabolic Engineering of Ashbya gossypii
Source: J Fungi (Basel). 2021 Oct 26;7(11):906. doi: 10.3390/jof7110906 (PMC8618306; doi:10.3390/jof7110906)
Supplement: Supplementary file 1 [file jof-07-00906-s001.zip › Table S2.pdf]

Table S2. Relevant sequences of the integrative cassettes

**Sequence name**	**Sequence (5'-3')**
ADR304W-left flank	AGTCCAAAACAGGGGAGCAGCGTGTAGAGCGCGCTAAGCCGGAGAACGTGGAGGAGGC<br>ACTGCGCCTGATTGAGGACCTGAAGTTCTTTCTTGCCACGGCGCCCGCGAACTGGCAGG<br>AAAACCAGGTGATCCGACGCTACTACTTGAACAACGACGAAGGCTTTGTGTCGTGCGTGT<br>TCTGGAACAATCTGTACTATATCACGGGGACGGACATTGTGCGATGCTGCGTGTACCGCA<br>TGCAGAAGTTCGGCCGCGAGGTTGTCGAACGCAAGAAATTCGAGGAGGGGATCTTCTCT<br>GATCTGCGGAACCTAAAGTGTGGGGTGGACGCCACGCTGGAGATGCCCAAGTCTGAGTT<br>TCTGTCGTTTCTGTACAAGAATCTGTGTCTGAAGACGCAGAAGAAGCAAAAGGTATTCTTC<br>TGGTTCAGCGTACCGCACGACAAGCTGTTTGCGGACGCTCTGGAGCGAGATTTGCGCCG<br>CGAGGTGGCCGGCCAGCCGTCTACCACCCGCGCCATAGCGGAGCCTGCGCTGACCTTT<br>CGATACGACGACCAGTCTGGAACGTCGCTCTACGATCAGGTCGTACAGCATGTGG
ADR304W-right flank	ACACTGATGACGATCCCAGCAACGAGGATCAGGATTCCGATAACGTCAACGAACCGATAG<br>AAGAACATGTTCCGATTTCAAATAATCCCAGAAATAGTAGTGGGATGGGTGCTTTTCCGCC<br>GCAAGATGCATACCTCGGCTACCAAGGCGTACCGCCAGCTGCCATGTATCCATATGTTAT<br>GCCAGATGTTTTCTACAATAATACATATCCAGGCGGCGATGAGTTGTATGAACAATGGATG<br>CATATGCAGTTTGGTCAACCACAGCAGAATGATCTGTTCATGCCCCCGCAAGCGCCATTT<br>ATGGGGCGCTCTTTCACTCCAATATACCGTACCACACCAACAAATCCATACATGGCCATTT<br>CTCCATATCAGTCAAAGCCTCCTACGTCTTCTACTGCAAAAAACTTTCCATTTTATCAGGG<br>ATATTATGGTCGACGGGGAAATATGCACTCATACCTACGCGGATTCCCCTCGATCCAAGC<br>CACACAGCCTTCTTCTGCCACGCGCATGCACTTTGTTAAGCACAACAGGGTAGGCACTCA<br>AAATGCGGCTGCATC
AGL034C-left flank	TGTGTCAGGTTCGATGGGCGGTATTTTGATCTAGCAACTAAAAGCTATATAAGGGCAACG<br>ATCCCTCATCTATTCAATCGTCTGGTTCGTATTCCCCACTCAATTCTAAGCTATTCATCAGT<br>CAACGAGAAGTTCTTAGACAAACAAGAAACATTATAGGAAATGAAAACCGCCAACGTTCTA<br>ACCGCTTTTGCTGCATCTACCGCCTTGTCCGCTAACCCAGCCAGTGCATTCATCACAAAC<br>GGTGCCGGAGCACCTCAAGTGGATGACAATCCAAAAGACGCCTGCTACGTAGCCGAGTT<br>CCCACAAGCCGGGAGCGACAAAGTAGCAGGTTACGTTACATTCACAGCATATGAAGGCAT<br>GACCAAGGTCAGCGTCGACTTGGCCACTGTGGTAAATCCAGAGGACGAAGGATTCTCCT<br>ACCACGTTCACGAAAAGCCAATTGAATCGGGAGATGAGTGTGCATGTTACGGAAACGGTG<br>ACGAACTCGACCCATACACTGGCATTGCTAGTTGCTCAGATGTCAAGGACAAATCTCTAT<br>GTAAGGTCGGTGACCTAAGTGGCAAATACGGGAACATAGAGTCCAATGTGTTCAAAGCGA<br>AATATTTGGACCCATATCTAGGGCTATACAGCTCATCCCCAAGTTTTATTGGAGGTCGCTC<br>CCTAACAATACACAACGGCGACAAGAGGCTAGCGTGT
AGL034C-right flank	GCCTGAGGAGCAGCCTGAAGACAAGCCTGAAGACAAGCCAGAGGAACCAGAGGACAAG<br>CCAGAGGAACCAGAGGAGCAGCCAGAAGGACCTGGTGTTCCAGCTCCAAAGGAACCTGA<br>GACTCCAGAAGGCGAAACCCCAGAAGGTCCAACCGGTCCAGGTGTCCCAGCTCCAGAGG<br>CTCCAGAGGCTCCAGAAGCTCCAGAAGCTCCAGAGGCACCAGAAGCTCCAGAAGGCCCA<br>ACCGGTCCAGGTGTCCCAGCTCCAGAGGCTCCAGCTCCAGAGGCCCCAGAAGTTCCAGA<br>AGGCCCAACCGGTCCAGGTGTCCCAGCTCCAGAGGCTCCAGCAGCTCCAGAGGCTCCA<br>GAGGTTCCAGAGGCTCCAGAAGCTCCAGAGGCTCCAGAGGCTCCAGAGGCTCCAGAGG<br>CTCCAGAGGCTCCAGAGGTTCCAGAGGCTCCAGAGGCTCCAGAGGCTCCAAGTGTCCCA<br>TCACCCGCTCCAGAAGTTCCAAGTGGCCCAGGTGTTCCTGCACCAGCTCCTACCTCTGTT<br>CCAGACGGAAGACCTGCCCCAGTCATCCCAT
Renilla luciferase from plasmid pRL-SV40 (NCBI accession number AF025845.2)	ATGACTTCGAAAGTTTATGATCCAGAACAAAGGAAACGGATGATAACTGGTCCGCAGTGG<br>TGGGCCAGATGTAAACAAATGAATGTTCTTGATTCATTTATTAATTATTATGATTCAGAAAA<br>ACATGCAGAAAATGCTGTTATTTTTTTACATGGTAACGCGGCCTCTTCTTATTTATGGCGA<br>CATGTTGTGCCACATATTGAGCCAGTAGCGCGGTGTATTATACCAGACCTTATTGGTATG<br>GGCAAATCAGGCAAATCTGGTAATGGTTCTTATAGGTTACTTGATCATTACAAATATCTTAC<br>TGCATGGTTTGAACTTCTTAATTTACCAAAGAAGATCATTTTTGTCGGCCATGATTGGGGT<br>GCTTGTTTGGCATTTCATTATAGCTATGAGCATCAAGATAAGATCAAAGCAATAGTTCACG<br>CTGAAAGTGTAGTAGATGTGATTGAATCATGGGATGAATGGCCTGATATTGAAGAAGATAT<br>TGCGTTGATCAAATCTGAAGAAGGAGAAAAAATGGTTTTGGAGAATAACTTCTTCGTGGAA<br>ACCATGTTGCCATCAAAAATCATGAGAAAGTTAGAACCAGAAGAATTTGCAGCATATCTTG<br>AACCATTCAAAGAGAAAGGTGAAGTTCGTCGTCCAACATTATCATGGCCTCGTGAAATCC<br>CGTTAGTAAAAGGTGGTAAACCTGACGTTGTACAAATTGTTAGGAATTATAATGCTTATCTA<br>CGTGCAAGTGATGATTTACCAAAAATGTTTATTGAATCGGACCCAGGATTCTTTTCCAATG<br>CTATTGTTGAAGGTGCCAAGAAGTTTCCTAATACTGAATTTGTCAAAGTAAAAGGTCTTCAT

	TTTTCGCAAGAAGATGCACCTGATGAAATGGGAAAATATATCAAATCGTTCGTTGAGCGAG<br>TTCTCAAAAATGAACAATAG
Firefly luciferase from plasmid pRL-SV40 (NCBI accession number AF025845.2)	ATGGAAGACGCCAAAAACATAAAGAAAGGCCCGGCGCCATTCTATCCTCTAGAGGATGGA<br>ACCGCTGGAGAGCAACTGCATAAGGCTATGAAGAGATACGCCCTGGTTCCTGGAACAATT<br>GCTTTTACAGATGCACATATCGAGGTGAACATCACGTACGCGGAATACTTCGAAATGTCC<br>GTTCGGTTGGCAGAAGCTATGAAACGATATGGGCTGAATACAAATCACAGAATCGTCGTA<br>TGCAGTGAAAACTCTCTTCAATTCTTTATGCCGGTGTTGGGCGCGTTATTTATCGGAGTTG<br>CAGTTGCGCCCGCGAACGACATTTATAATGAACGTGAATTGCTCAACAGTATGAACATTTC<br>GCAGCCTACCGTAGTGTTTGTTTCCAAAAAGGGGTTGCAAAAAATTTTGAACGTGCAAAAA<br>AAATTACCAATAATCCAGAAAATTATTATCATGGATTCTAAAACGGATTACCAGGGATTTCA<br>GTCGATGTACACGTTCGTCACATCTCATCTACCTCCCGGTTTTAATGAATACGATTTTGTA<br>CCAGAGTCCTTTGATCGTGACAAAACAATTGCACTGATAATGAATTCCTCTGGATCTACTG<br>GGTTACCTAAGGGTGTGGCCCTTCCGCATAGAACTGCCTGCGTCAGATTCTCGCATGCCA<br>GAGATCCTATTTTTGGCAATCAAATCATTCCGGATACTGCGATTTTAAGTGTTGTTCCATTC<br>CATCACGGTTTTGGAATGTTTACTACACTCGGATATTTGATATGTGGATTTCGAGTCGTCT<br>TAATGTATAGATTTGAAGAGGAGCTGTTTTTACGATCCCTTCAGGATTACAAAATTCAAAGT<br>GCGTTGCTAGTACCAACCCTATTTTCATTCTTCGCCAAAAGCACTCTGATTGACAAATACG<br>ATTTATCTAATTTACACGAAATTGCTTCTGGGGGCGCACCTCTTTCGAAAGAAGTCGGGGA<br>AGCGGTTGCAAAACGCTTCCATCTTCCAGGGATACGACAAGGATATGGGCTCACTGAGAC<br>TACATCAGCTATTCTGATTACACCCGAGGGGGATGATAAACCGGGCGCGGTCGGTAAAGT<br>TGTTCCATTTTTTGAAGCGAAGGTTGTGGATCTGGATACCGGGAAAACGCTGGGCGTTAA<br>TCAGAGAGGCGAATTATGTGTCAGAGGACCTATGATTATGTCCGGTTATGTAAACAATCC<br>GGAAGCGACCAACGCCTTGATTGACAAGGATGGATGGCTACATTCTGGAGACATAGCTTA<br>CTGGGACGAAGACGAACACTTCTTCATAGTTGACCGCTTGAAGTCTTTAATTAAATACAAA<br>GGATATCAGGTGGCCCCCGCTGAATTGGAATCGATATTGTTACAACACCCCAACATCTTC<br>GACGCGGGCGTGGCAGGTCTTCCCGACGATGACGCCGGTGAACTTCCCGCCGCCGTTG<br>TTGTTTTGGAGCACGGAAAGACGATGACGGAAAAAGAGATCGTGGATTACGTCGCCAGTC<br>AAGTAACAACCGCGAAAAAGTTGCGCGGAGGAGTTGTGTTTGTGGACGAAGTACCGAAA<br>GGTCTTACCGGAAAACTCGACGCAAGAAAAATCAGAGAGATCCTCATAAAGGCCAAGAAG<br>GGCGGAAAGTCCAAATTGTAG
