# Supplementary material for: New Promoters for Metabolic Engineering of Ashbya gossypii
Source: J Fungi (Basel). 2021 Oct 26;7(11):906. doi: 10.3390/jof7110906 (PMC8618306; doi:10.3390/jof7110906)
Supplement: Supplementary file 1 [file jof-07-00906-s001.zip › Table S3.pdf]

Table S3. List of primers used in this study.

Primer	Sequence*	Purpose
Firefly-SapD-fw	5’-GAAGAGGAGCTGTTTTTACGATCCCTTCAGGATTA-3’	Firefly cloning
Firefly-SapD-rv	5’-AACAGCTCCTCTTCAAATCTATACATTAAGACGACTCG-3’	Firefly cloning
Firefly-GATG-fw	5’-TTTTTGGTCTCA**GATG**GAAGACGCCAAAAACATAA-3’	Firefly cloning
Firefly-TAGT-rv	5’-AAAAAGGTCTCA**ACTA**CAATTTGGACTTTCCGCCC-3’	Firefly cloning
Renilla-GATG-fw	5’-TTTTTGGTCTCA**GATG**ACTTCGAAAGTTTATGATCCAGA-3’	Renilla cloning
Renilla-TAGT-rv	5’-AAAAAGGTCTCA**ACTA**TTGTTCATTTTTGAGAACTCGCTC-3’	Renilla cloning
GGTC-CCW12p-fw	5’-TTTTTGGTCTCG**GGTC**GATCCTCGAGATCGGCCG-3’	P_{CCW12} cloning
GATG-CCW12p-rv	5’-AAAAAGGTCTCA**CATC**TTCGGTGTAAGGAAGCGAGT-3’	P_{CCW12} cloning
GGTC-CWP1p-fw	5’-TTTTTGGTCTCG**GGTC**TACCTTCGGAAGTGGTCGCA-3’	P_{CWP1} cloning
GATG-CWP1p-rv	5’-AAAAGGTCTCA**CATC**TTGAATTGTTGGATGTTTGTGTGAC-3’	P_{CWP1} cloning
GGTC-TSA1p-fw	5’-TTTTGGTCTCG**GGTC**ATCATGGCACCTATCTAGTT-3’	P_{TSA1} cloning
GATG-TSA1p-rv	5’-AAAAAGGTCTCA**CATC**TGTAGAATTGCTGTTTACAGA-3’	P_{TSA1} cloning
GGTC-AFR038Wp-fw	5’-TTTTTGGTCTCG**GGTC**GGACACGTACCTTTGGGATA-3’	$P_{AFR038W}$ cloning
GATG-AFR038Wp-rv	5’-AAAAGGTCTCA**CATC**CTTTGTTTTGCGCGATGACT-3’	$P_{AFR038W}$ cloning
GGTC-CDA2p-fw	5’-TTTTGGTCTCG**GGTC**GGCTCGCGCCAGGCACAGGCAACGT-3’	P_{CDA2} cloning
GATG-CDA2p-rv	5’-AAAAAGGTCTCA**CATC**GCTGCTCCACGGTCGCT-3'	P_{CDA2} cloning
GGTC-TMA10p-fw	5’-TTTTGGTCTCG**GGTC**AACCCGGGGCTTCTGAAATTC-3’	P_{TMA10} cloning
GATG-TMA10p-rv	5’-AAAAAGGTCTCA**CATC**TGTGTCCTTAGCTGCTGAACT-3’	P_{TMA10} cloning
GGTC-SED1p-fw	5’-TTTTGGTCTCG**GGTC**CCGCATAGTCTAATGAAGATTCTTT-3’	P_{SED1} cloning
GATG-SED1p-rv	5’-AAAAAGGTCTCA**CATC**TGACTTTATGAAAGCGATAGGATG-3’	P_{SED1} cloning
GGTC-AGL366p-fw	5’-TTTTGGTCTCG**GGTC**TTACTGATACCAGTCCACATCGGT-3’	$P_{AGL366C}$ cloning
GATG-AGL366p-rv	5’-AAAGGTCTCA**CATC**CGCGCAATCTAAAGATTCGAAACA-3’	$P_{AGL366C}$ cloning
GGTC-HSP26p-fw	5’-TTTTTGGTCTCG**GGTC**CGTCTGCCCGTGGTGACC-3’	P_{HSP26} cloning
GATG-HSP26p-rv	5’-AAAAAGGTCTCA**CATC**ATTGCTGTTAGGCTGAGTTGC-3’	P_{HSP26} cloning
GGTC-PFS1p-fw	5’-TTGGTCTCG**GGTC**CTTTTTCCATTTTTATAACTCTAACAC-3’	P_{PFS1} cloning
GATG-PFS1p-rv	5’-AAAAAGGTCTCA**CATC**CTGGAAGGAGGTGCTATACT-3’	P_{PFS1} cloning
kanB	5’-CTGCAGCGAGGAGCCGTAAT-3’	analytical PCR
AGL034C-a	5’-CAGCTCATCACAACGCTTATCCA-3’	analytical PCR
ADR304-a	5’-TGTCGGTGCTCAAGACAGAC-3’	analytical PCR

loxPMK-P_{GPD1}-MSN2-ins5	5'-AGCAGCAGATAAGGTGAGAACAAAAGCGTCCGGATAGCGAAGCAAGCTATAACGGTATAGAGCAGCCGTACACTAAGTGTCGGATCCCCGGGTTAATTAA-3'	*MSN2*-expression
$P_{AGL366C}$-MSN2-ins3	5'-CCTGTCAGACCCATGGCCGTCGCGCCCTGCCCTAACTCAGCGCCAAGAAGATACGCTGTGTATTCTGCAGAAGTCATCGCGCAATCTAAAGATTCGAAAC-3'	*MSN2*-expression
P_{SED1}-MSN2-ins3	5'-TGTCAGACCCATGGCCGTCGCGCCCTGCCCTAACTCAGCGCCAAGAAGATACGCTGTGTATTCTGCAGAAGTCATCTGACTTTATGAAAGCGATAGGATG-3'	*MSN2*-expression
$P_{AFR038W}$-MSN2-ins3	5'-GCCCCTGTCAGACCCATGGCCGTCGCGCCCTGCCCTAACTCAGCGCCAAGAAGATACGCTGTGTATTCTGCAGAAGTCATCTTTGTTTTGCGCGATGACT-3'	*MSN2*-expression
P_{TSA1}-MSN2-ins3	5'-GTCAGACCCATGGCCGTCGCGCCCTGCCCTAACTCAGCGCCAAGAAGATACGCTGTGTATTCTGCAGAAGTCATTGTAGAATTGCTGTTTACAGAGTTTG-3'	*MSN2*-expression
MSN2-a	5'-CAAGTCCCGCTACAGAACCA-3'	analytical PCR
UBC6-qPCR-fw	5'-TCCGCCCGCGATCAGGATG-3'	qRT-PCR
UBC6-qPCR-rv	5'-CTTGCGCTTCGTGGAGTCCGTAGA-3'	qRT-PCR
MSN2-qPCR-fw	5'-CAGGCGGAGCAGCAGTTCAT-3'	qRT-PCR
MSN2-qPCR-rv	5'-CCCGATACGCCCGATAGCAT-3'	qRT-PCR

**Bsa*I sites are underlined. 4-nt overhangs are indicated in bold
