# Supplementary material for: New Promoters for Metabolic Engineering of Ashbya gossypii
Source: J Fungi (Basel). 2021 Oct 26;7(11):906. doi: 10.3390/jof7110906 (PMC8618306; doi:10.3390/jof7110906)
Supplement: Supplementary file 1 [file jof-07-00906-s001.zip › Table S4.pdf]

Table S4. Intergenic sequences used for the promoter analyses

Gene name	Sequence (5'-3')
AGR049W (CCW12)<br>GenBank: AAS54538.1	GATCCTCGAGATCGGCCGGGTAACCCGCGGCCGCTTCTATTTTAGTATTCATATCTCAAGCA<br>CATCCATTCCGGCCGTTTGGGGGCGCCGCCGCACTCGTGTCCATTCCTACCGTGGCACTTA<br>GGGCTATCCTGTCGGAGCGCCCCGCCGACCGCCTTATCGGCACCAAAAGTAGAAGCCCCGG<br>CCCCGCGTGGCTCAGACTCACCATCGGTGCTATTTACTTTTCGATCAGATCGCGGCGCGCGG<br>TGGCCGGCATTTCCGGAAGCGGCCACGGAGCAGAGGTGGCGCATTCGAATCGCATACGTCT<br>TCGCCACGCCGGAAAAAAAATTTTCGGCTATATAAGGAGAGGCGGCCGTCTTGCTGCAGGCA<br>GTTTCACTTTCTCTAAAACCAAAGAACATCGATTTCTTTAGTCACTCGCTTCCTTACACCGAA
AFR505C (TMA10)<br>GenBank: AAS53876.1	AACCCGGGGCTTCTGAAATTCTATAGCTCTTTAGTGTAATCAATTACGGTTTCATAGTCATCCG<br>GCCCCTCGATGACCTGGCAAGGGGGGTCACGTGAGCACAACCTGCGGATAGCACAGCCTTG<br>TGCTCTGCCCCCTAAGTTTCCATTAGAGTCACCCCCGCTGGGTATATAAACTGGAGAAAGCG<br>GTTCCTCTGACTGATGGAGCTAACAAGACACGGTTCAGCAGCTAGCAAGAAGTTCAGCAGCT<br>AAGGACACA
ACR272C (CWP1)<br>GenBank: AAS51498.1	TACCTTCGGAAGTGGTCGCACACGTCTCTACCAGACGGGTACAGATGAACAGCCTGTGCCGC<br>CACTTCGAAACAAAGGGACCAGTAAAGCCTTGCTGGGGCGGCTACAGTGCGGAAGCCATCT<br>CGAGGGCTAAATTTGGATCCAAACAAAGAGCTCATAGACAGGGATGAGAATGAATACCTCAC<br>GAGCAGCTCCACCGCATTGCACCCCTGCGAAAATGTTACTTGTAAGCGATTATATCAGCATG<br>GTTACTGTTCCAGTGTTTTGAGCGGCACGTGCGGCCCGGCGCAGTACCTGAAATTGCCATAG<br>GGTGACAGATAGATGTAACATAACTTCCGCCCATGTAATAGCAGGTAGCAAGCAATATTGTGG<br>CTTAATTAGCGCCGAAAGGCACGGCAGCAGAGCCAAAATCTCACTTAGTATTCCTTTGTAATT<br>GGCAGAGCAATTAAACACTTTCAGACGAAAGCGCCAAGTAACTCGGTTCCGTGGAAGACCAA<br>TTGGCTCAGATTGCCTTCGTAGGATACTTCCGCCATCTTGATGTGTTTGCAATAGCGCACGAA<br>AGATTTACCATGCCATGTATCGACATCGTTGTGTTTCAACGTGATGTGAAGGCACCCGTCCAC<br>ACCTACAAGATCGCAATGCAACTGCTTTCTTTATTTAGAACTTCTCATTGCATCGAAGGGCCT<br>GCTTAAGGACGGGACATAAATTGCGTCATGGTCCCAAGTATACAGCTGACAAAGGCCACCCT<br>AGGGATGCCGCAGCCGCCAACTTATGGTTAAGCCCCATTTGTTGGCATTATACGAGGTGCTA<br>TTTTTAATGAGTGCCACGACAGGGCCCTTTAGGAGTAAGTCCCAAACGTATATAAAGCGAGAG<br>ATGAATCAACTCTAGTCTATCGCTTAGCAGACTCGGTAAGCAGTCTTTGACAGTCACACAAAC<br>ATCCAACAATTCAA
AER312W (TSA1)<br>GenBank: AAS52992.1	ATCATGGCACCTATCTAGTTCAATACGCTTATGCTCCGCCCGCAATTCCGTTTATCTCCGCTC<br>CGCTTCTCTGCGCTTCTGGAAGGCTTCGGGCGCTCGGGCGGCCGGGTAATGACCCGGGTG<br>GCAACGGCGCCGCATTAGGGGGGTTGACTGACTGATAACCAAAAAAATATAAAGAAGGCGG<br>CAGATGCGATCAGATTTTGTGGCTGGCCCCTTCATACCTCGCCACAAACTCTGTAAACAGCAA<br>TTCTACA
AER031C (GPD)<br>GenBank: AAS52715.2	GTCTGGGTGCACGACACCTGACCTCCGCCCCGCGGGCTTCCTGTTTTCGCCGGGCGCGGCA<br>CATGGTGCGGCTTCCTCCGACAGGAAGCCGGGCCGCCGGACGCGCACGTCAGAGGCGTCA<br>CCAGGGCAAATGGGTGGAAGCGAAGGGAACTACGACGAACGGTCAGCACCCCTGGGGCCC<br>CCACGCTCGCACCACAGCCGCTGCGCGTGGGCGTGAAAAATTTTACCTGCGGGCTCTCCTTA<br>CGATCTCCTATTTTATTTCCTGGGGGGCAGTCGAAATCTATATAAGAGGGCCCCGGGACGCA<br>CAACGGGAGGACTCTGGTGGAGCGACCAGGAGTTTGAATTAATTCAGTCCACACATACACAC<br>CGCACA
AGL366C<br>GenBank: AAS54125.1	TTACTGATACCAGTCCACATCGGTAGTTTTGTTAATAGCACGCTAATTCTAAAAGCATGGCGAT<br>GTTTCAAATTCCTCTTAATTCTTCGATAGGAGAAGCTGTTCTATAGTTTTGTGTTCTAGTGTACT<br>CGTTCCGCAAATATATATTTCTAATTTAAGCACAACGGTAAATACTATGTCCTTTATTTCAGATG<br>CAAGAACTGGACCATCTGAATGGTGCGTCCGAAATTGCAGACTCTGTGGAGACACCAATTCT<br>GTTATAGCTCGACCGGCGCAGACATCTGTCCAACAATTGGTCGTCGATCAATCATTAAATGAC<br>AAGCTTAGCAGATAATCCTTGGTGTCAGTAGTAAGTCGCATGCCGCAAGGAACACGGAGACG<br>TGCGGCCTTGTTTGATTACGTCTCGGCATTTTTTCCCTTTATAGAAACGAAACTCCGCTAAGAT<br>GCCTCATGCTCTATAGTTAGTAACCAACAGTGGGGTTGGTTATGTACGGCATTTAATCGACAT<br>ACTGAAAATTACCCCTTATGGATTAAAGATGAGGCGTTAAAGATTTACCTTTGACACCGGAGA<br>TGTTCGATTCTTTTGTATATAATAAGTGTAAGGAATCATGGCATTTTGGCTGCCAATGACTAAA<br>TACTACAGTTATCTGTTTCGAATCTTTAGATTGCGCG
AGR138W (SED1)	CCGCATAGTCTAATGAAGATTCTTTTATTAGAAACAAAGAGCCCTTTCCTTGCCTTTGGGGGA<br>TGTCCCGTTTCCATAGGTGTGGCTGGCAGGGTGTGCCCCTGTAGGGTGGCTTTAGCGGGCG<br>GCATGATGTACCGCGCGGCGGGTGGGCACGACACCGGGGATTACTACAGATGGACAAAGTT<br>CCTCAAGGGCATCGCTGTACGCGGGAGTACTACCGCTCTGGGCCGTGCCTGGGAGCGGGA

GenBank:<br>AAS54628.1	AGCATGGAACAGAGCGGAAAATCGGATAGCACGGGCGCGTCGAGAACGCGCGGGAACGGC<br>CCAGCAGGCATTACTGGGGAGCTAGGGACCAGGGCGGGTGCTTCTGTTCTCGGTAGGGGC<br>GGATGCGTGGGGGTTTCGGCTTAGTTGTCTGTGGACAAGATTTGCCGTAGAGACGCAGATG<br>GTGATATTAACTAGACAGGTAATTAATTGCCTGGCAGGGATCGGAAGGTTGGCCGATCGAGG<br>CGTAAGGGTGCGGGAGTTATTTATGGAAATTTCGAGTGCTTGCGCCACAAGCGATTCGCTGG<br>GGAAGTTCTATTTTAGCAGCGACTTTGATGGTGTCGCCAGTATATAAGAAGCATTGGCGAACA<br>AAGAATCTGTATGGTGTTCCTTGTTCCTCACCAAGCCAATTGATCAGATCTGAGTTTGATACTT<br>TAGCCGATTCATCCTATCGCTTTCATAAAGTCAG
ADL036C (CDA2)<br>GenBank:<br>AAS51884.1	GGCTCGCGCCAGGCACAGGCAACGTAGGGTCCGCGCGCGACGGCGGGGCTGCCGCAGCG<br>CTGCGGCACACGGCGACGGTGACTGGTGGCAGAGCATGGCGACGGCGGGCAGTACCAGCA<br>CAGACGGCGACAGCCGGCGGGCAGCGCAGATACGACGGCGGGAGGGGGCAGTGCAGCGG<br>ACGGCGACGGGCGGACAGAGCACAAAGACAGACTACGGGGGCTATAAGACACCGGGCGGG<br>GCAGGTGTGTTCGCGGTGCTGGAAGGGAGCAGTGTCTACGGATAGCGTATCGAGAAGAACA<br>CAGACACAGAGCGACCGTGGAGCAGC
AGR408W (HSP26)<br>GenBank:<br>ADJ41746.1	CGTCTGCCCGTGGTGACCGGATGATGGATCTGCTTTCTGCCAATGCACGTGGCAAATGCACG<br>TGCCCGACCGGGTGCTGAATCAGAGCGTTGGAGAATGCAGACGGGCACTTGCAGTGGCCTA<br>CAATTTACGGCAGGCACGCGGCGAGGTATATCCTGAGAGGTTTTCGAGACTTGCTCGTAGCT<br>ATTGCTTTTCACGGGGCTAGACCGGCCATTTGTTGGCGGGATGCGCAGCCACGCAGGCTGA<br>CGCTTCGGCCCACCTAAACACTAAAGCACTACAGGCCAGCGACTACGCCCAGTGTAGCCAAG<br>ATGGTGACAGATGGTGACAGATTCTCAGCGGCAGCAATCGGTTCCGAGCGCTAGAGCGCAG<br>TAACGGCCAACGGCTTGCTGCCGGGTGACAGTATGCATCTGCACCGGAGGCGGGGGCGAT<br>GCTGACCGCTGGCCGGCACGCTGTAGAGGGGCGCAGCCAGCCGTCAGCCGATACTCTATCC<br>CGGATCGACCCAGACGGCGCAGGAATCCCCGGCGCCCAACATATTGGTGTCGAAGGGTTTG<br>GGTTTTCACGTGTACTGTATGTGGCAGGCGCTCACTGCTACTTTTTCCGTGTAGGCGGCTTTC<br>TCGCGGGGATGGCCAACTCCGTAAACTACGCGTTCGTGCGGTGGTCCGGTGCAGCTACCTT<br>GTGAGCAGATACACACCATATTAAATGCCCAAACCGACGGCACTACCCTATGCCTTCAGGGC<br>TACGCCCGTAGTTGTTTTTGTACCCAGTATTTAGTCCAGTTGCACCTGCCCACATATAGTCAC<br>GTGATTGTGATCGACTAGAAAAATCTATAATGTTCCAGCTCCGATGTCAGCGTCATTGTTCTG<br>CCGCACCAGGGGTAGAACCTCCAGCGTATAAATTGAAGTGCAAGAACAAGCTCAGTTCAGTT<br>CCTAGAGCGATCATAAGCAAGAGTTCACCAAAGAGAAATAGTGTTCAACAAATAGCAACTCAG<br>CCTAACAGCAAT
AFR038W<br>GenBank:<br>AAS53409.1	GGACACGTACCTTTGGGATACGCCCGCCCCTAGCATGCTGCAGCAGGCCAGCGCGACGCTG<br>GCAGTCCGCAGCTATTTTAATGCGTCTGCAGTAAGCAACGGCGCCCTTAATATGGCCTGTTA<br>CCCGGTGTTTGAGATGTATTTAAAGGTCTACCCATCGCCTCTGGTAGCGCAAGTCCGGTAGT<br>CATCGCGCAAAACAAAG
AFR132C (PFS1)<br>GenBank:<br>AAS53503.1	CTTTTTCCATTTTTATAACTCTAACACCGACGCTGACGCTGACGCTCCACAGGAGCAGAGCGT<br>CAAGAGGCGGTATTCTTTGTACACAAATAGAATGAGCTAGAATATAAAAGACCGTAGCTGTCT<br>CAAGGCAATTCAGAATACTATATACAAAAAAGTATAGCACCTCCTTCCAG
