# Supplementary figures and images for: New Promoters for Metabolic Engineering of Ashbya gossypii
Source: J Fungi (Basel). 2021 Oct 26;7(11):906. doi: 10.3390/jof7110906 (PMC8618306; doi:10.3390/jof7110906)

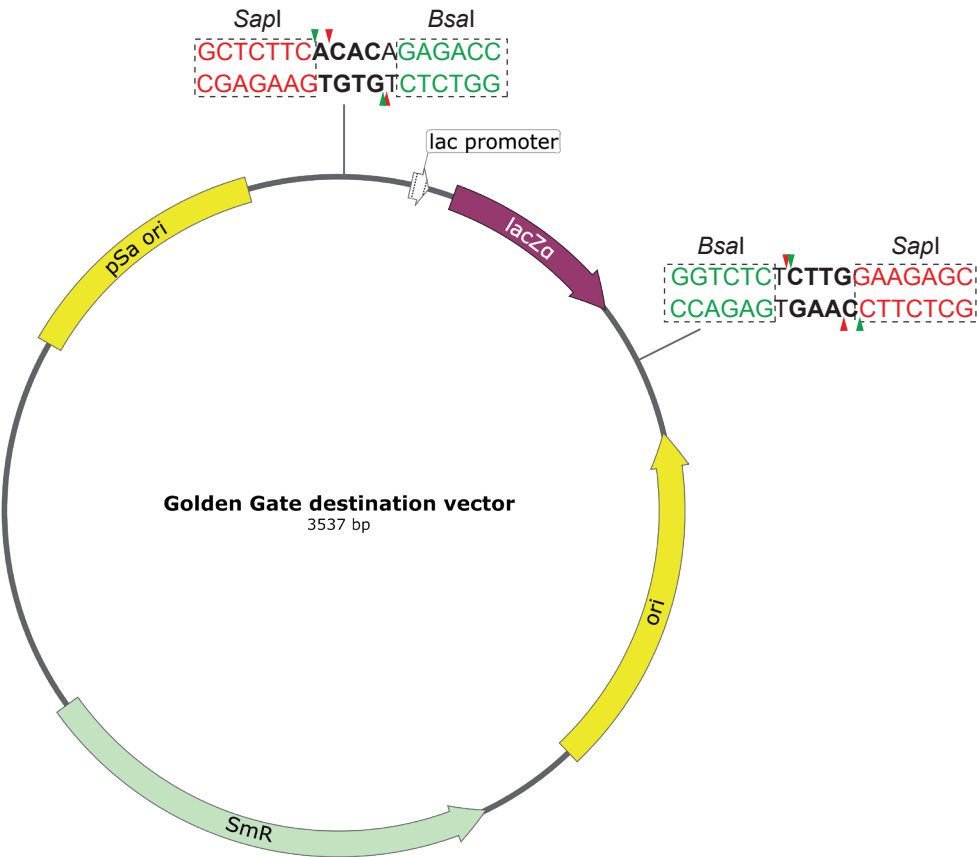

Supplement: Supplementary file 1 [file jof-07-00906-s001.zip › Figure S1.pdf]
